# Is increased positive end-expiratory pressure the culprit? Autoresuscitation in a 44-year-old man after prolonged cardiopulmonary resuscitation: a case report

**DOI:** 10.1186/s13256-016-1148-4

**Published:** 2016-12-20

**Authors:** Henning Hagmann, Katrin Oelmann, Robert Stangl, Guido Michels

**Affiliations:** 1 Department II of Internal Medicine and Center for Molecular Medicine Cologne, University of Cologne, Cologne, Germany; 2 Fire Department and Emergency Medical Service Cologne, Cologne, Germany; 3 Department III of Internal Medicine, Heart Center of the University of Cologne, Cologne, Germany; 4 Department II of Internal Medicine, University Hospital Cologne, Kerpener Str. 62, 50937 Cologne, Germany

**Keywords:** Autoresuscitation, Lazarus phenomenon, Advanced cardiac life support, Emergency ultrasound, Capnometry

## Abstract

**Background:**

The phenomenon of autoresuscitation is rare, yet it is known to most emergency physicians. However, the pathophysiology of the delayed return of spontaneous circulation remains enigmatic. Among other causes hyperinflation of the lungs and excessively high positive end-expiratory pressure have been suggested, but reports including cardiopulmonary monitoring during cardiopulmonary resuscitation are scarce to support this hypothesis.

**Case presentation:**

We report a case of autoresuscitation in a 44-year-old white man after 80 minutes of advanced cardiac life support accompanied by continuous capnometry and repeated evaluation by ultrasound and echocardiography. After prolonged cardiopulmonary resuscitation, refractory electromechanical dissociation on electrocardiogram and ventricular akinesis were recorded. In addition, a precipitous drop in end-tidal partial pressure of carbon dioxide was noted and cardiopulmonary resuscitation was discontinued. Five minutes after withdrawal of all supportive measures his breathing resumed and a perfusing rhythm ensued.

**Conclusions:**

Understanding the underlying pathophysiology of autoresuscitation is hampered by a lack of reports including extensive cardiopulmonary monitoring during cardiopulmonary resuscitation in a preclinical setting. In this case, continuous capnometry was combined with repetitive ultrasound evaluation, which ruled out most assumed causes of autoresuscitation. Our observation of a rapid decline in end-tidal partial pressure of carbon dioxide supports the hypothesis of increased intrathoracic pressure. Continuous capnometry can be performed easily during cardiopulmonary resuscitation, also in a preclinical setting. Knowledge of the pathophysiologic mechanisms may lead to facile interventions to be incorporated into cardiopulmonary resuscitation algorithms. A drop in end-tidal partial pressure of carbon dioxide, for example, might prompt disconnection of the ventilation to allow left ventricular filling. Further reports and research on this topic are encouraged.

## Background

The delayed return of spontaneous circulation (ROSC) after cessation of cardiopulmonary resuscitation (CPR) measures, also known as autoresuscitation, is known to most emergency physicians and intensive care specialists [1]. Cases in adults and children are reported regularly in which spontaneous circulation ensued several minutes after all supportive measures were withdrawn [2–9]. The pathophysiology behind the phenomenon of autoresuscitation remains nebulous. Several hypotheses have been generated to explain the unexpected recovery of spontaneous circulation [10]. Among these are alterations in the electrolyte balance, notably hypokalemia and hyperkalemia, as well as ventilation-associated causes like the generation of high positive end-expiratory pressure (PEEP), which impairs venous return and ventricular filling [11]. In addition, delayed drug effects due to unfavorable milieu (that is, acidosis or alkalosis) or impaired drug delivery via peripheral lines or intraosseus access sites have been held accountable for primarily unsuccessful CPR and delayed ROSC. Elucidation of the pathophysiologic processes of autoresuscitation may be hindered by the limited reports including invasive cardiopulmonary monitoring, blood gas analysis, and imaging studies during CPR. We report a case of autoresuscitation in a 44-year-old white man after 80 minutes of CPR according to the current European Resuscitation Council (ERC) guidelines including capnometry and focused cardiac/emergency ultrasound in a preclinical setting. After prolonged CPR a drop in end-tidal partial pressure of carbon dioxide (p_et_CO_2_) was noted; this might indicate increased intrathoracic pressure inhibiting successful resuscitation. A case like this may help us to learn more about the underlying pathophysiology of autoresuscitation because CPR was accompanied by extended cardiopulmonary monitoring.

## Case presentation

**Fig. 1:**
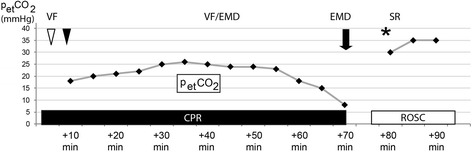
Serial end-tidal partial pressure of carbon dioxide readings during cardiopulmonary resuscitation and after return of spontaneous circulation. During automated cardiopulmonary resuscitation end-tidal partial pressure of carbon dioxide values of up to 26 mmHg were recorded. After 60 minutes of cardiopulmonary resuscitation end-tidal partial pressure of carbon dioxide rapidly dropped to values of less than 10 mmHg. *White arrow head* indicates arrival of emergency physician; *black arrow head* indicates arrival of emergency medicine attending physician. Time of withdrawal of all supportive measures is indicated by the *black arrow*. Resumption of monitoring is marked by *asterisk*. The respective electrocardiogram (ECG)-rhythm is specified in the top lane. The duration of cardiopulmonary resuscitation is marked with the *black bar*. Return of spontaneous circulation is indicated by the *white bar. CPR* cardiopulmonary resuscitation, *EMD* electromechanical dissociation, *p*_*et*_ *CO*_*2*_ end-tidal partial pressure of carbon dioxide, *ROSC* return of spontaneous circulation, *SR* sinus rhythm, *VF* ventricular fibrillation

A 44-year-old white man with a past medical history of viral myocarditis, reduced left ventricular function, and continuous beta-blocker therapy, collapsed on the street. Lay basic life support was initiated in less than 2 minutes by bystanders. Paramedics proceeded with CPR according to the current guidelines with a compression-ventilation ratio of 30:2 after approximately 5 minutes. An electrocardiogram (ECG) showed ventricular fibrillation (VF) as the primary rhythm which was refractory to defibrillations. An emergency physician reached the location 8 minutes after the initial incident. Administration of epinephrine (20 mg total), amiodarone (450 mg total), magnesium-sulfate (2 g), and sodium bicarbonate (100 ml of 8.4 %) via a peripheral venous route and repeated defibrillations at 200 to 360 joules did not restore a viable heart rhythm. An endotracheal tube (ID 8.0 mm) was inserted without complications and ventilation was continued with a resuscitation bag. Subsequently the patient was mechanically ventilated with a mobile respirator in a volume controlled mode (continuous positive pressure ventilation; CPPV) tidal volume 10 ml/kilogram bodyweight, PEEP 5 cmH_2_O, and fraction of inspired oxygen (FiO_2_) 1.0 at 12 breaths/minute. A second emergency physician (attending) equipped with an automated CPR device (LUCAS 2, Physio-Control, Redmond, USA) and an ultrasound unit was ordered to the site. Capnometry after endotracheal intubation showed a p_et_CO_2_ of 20 to 25 mmHg (Fig. 1). Pleural effusion, pulmonary-venous edema, and pneumothorax could be ruled out by lung ultrasound. Cardiac tamponade and burden on the right side of his heart were ruled out by focused cardiac ultrasound. His left ventricle was globally akinetic, his inferior vena cava (IVC) was not distended. CPR was continued using the automated CPR device, providing a constant high quality CPR. The ECG showed intermittent VF and electromechanical dissociation (EMD). Due to his history of myocarditis a rhythmogenic cause of the out-of-hospital cardiac arrest was suggested. The option of empiric thrombolysis for potential myocardial ischemia was abandoned due to lack of evidence of acute myocardial infarction or acute pulmonary embolism, as well as suspected craniocerebral injury after his collapse, since facial and periorbital lacerations were noted. After 80 minutes of CPR persistent EMD was recorded at a rate of 25/minute. The patient had fixed and dilated pupils, p_et_CO_2_ had decreased to less than 10 mmHg, and left ventricular akinesis was unchanged. The team reached the unanimous decision to quit resuscitation efforts. The infusion of catecholamines, mechanical ventilation, and the automated CPR device were stopped. He remained on the ECG monitor according to current practice showing continuous slow EMD. The ventilator was disconnected from the endotracheal tube, the tube remained *in situ*. Five minutes after withdrawal of all supportive measures audible breathing resumed. A subtle bradycardic pulse was palpable at his carotid artery. QRS-complexes increased in frequency and appeared increasingly narrow. Capnometry showed a spontaneous increase of p_et_CO_2_ to 30 mmHg. Focused cardiac ultrasound showed coordinated ventricular activity with reduced ventricular output. Catecholamine support was restarted; he was ventilated and hypothermia initiated. Shortly after he had a systolic blood pressure of 130 mmHg, a p_et_CO_2_ of 35 mmHg, and a peripheral oxygen saturation of 99 % under FiO_2_ 0.5. The ECG showed a supraventricular rhythm followed by sinus rhythm. His pupils were round, isocoric, non-dilated, and reactive to light.

He was taken to the Heart Center of the University Hospital Cologne. Coronary artery disease was ruled out by coronary angiography including left ventriculography; emergency echocardiography revealed severely reduced left ventricular function with an ejection fraction of 25 to 30 % and global hypokinesis of his ventricular wall with a basal septal ventricular wall aneurysm. His right ventricle appeared narrow; his IVC was found to be collapsing on respiration >50 % indicating fluid responsiveness. He was admitted to our cardiac intensive care unit; mild therapeutic hypothermia was maintained using an intravascular cooling device (Cool Line catheter with CoolGard System). The pH value was 7.32 in the first arterial blood gas analysis; his serum electrolytes were not significantly altered, specifically serum potassium was only slightly reduced (3.1 mmol/l). Inflammatory markers were not increased; levels of neuron-specific enolase (NSE) were 81.1 μg/l (upper reference limit 16.4 μg/l). A chest X-ray showed no pathologic findings and a toxicology screen was negative for illicit drugs. After fluid resuscitation the catecholamine infusion could be tapered. A cranial computed tomography (CT) scan revealed diffuse cerebral edema and decreased cortical gray matter attenuation with loss of normal gray-white differentiation. His NSE had increased to 313.5 μg/l indicating poor neurologic outcome [12]. Bilateral absent cortical response on somatosensory evoked potential were recorded 3 days after the incident representing an unfavorable prognosis. According to the presumptive decision of the patient his therapy was limited to supportive measures.

## Discussion

This is a tragic case of primarily unsuccessful resuscitation and delayed ROSC. The phenomenon of autoresuscitation leaves many open questions. Most importantly, the etiology of spontaneous recovery of a perfusing rhythm after cessation of CPR needs to be resolved. Once the etiology of autoresuscitation is known, algorithms may be modified in order to avoid circumstances which preclude effective CPR.

To the best of our knowledge this is the first published case of autoresuscitation after prolonged CPR in a preclinical setting, with serial echocardiographic imaging and subsequent immediate cardiac catheter. 

In our case, CPR was accompanied by preclinical capnometry and focused cardiac/ emergency ultrasound. By these means most reversible causes for cardiac arrest could be ruled out. Although blood gas analysis could not be performed during the CPR in the preclinical setting, electrolyte disturbances or pH disturbances are highly unlikely in our patient, since the initial laboratory values on hospital admission showed serum pH 7.3, potassium 3.1 mmol/l, and normal blood urea nitrogen (BUN)/creatinine ratio. There was no evidence of preexisting renal failure, illicit drug use, or chronic pulmonary disease.

Remaining low cardiac activity and unobserved minimal vital signs, as suggested in some reports, is most unlikely in our case given complete ventricular akinesis on focused cardiac ultrasound, which was reconfirmed at the time of the decision to terminate CPR efforts [13].

Dynamic hyperinflation of the lungs and increased PEEP is proposed as another cause of autoresuscitation [10, 11]. Increased PEEP leads to increased intrathoracic pressure, which impedes venous return and ventricular filling. As an approximation to intrathoracic pressure intrinsic PEEP can be determined automatically by modern respirator machines. In a preclinical setting, however, high intrathoracic pressure is difficult to detect. Ventricular size will not necessarily be affected by increased intrathoracic pressure [14–17]. Distension or collapse of the superior vena cava and IVC largely depend on the patient’s fluid status and cannot be attributed to increased thoracic pressure and PEEP alone [17, 18]. Therefore focused cardiac/emergency ultrasound, even Doppler ultrasound, cannot reliably rule out increased intrathoracic pressure and increased PEEP during CPR.

Of interest, our patient presented with a precipitous drop in p_et_CO_2_ to 10 mmHg after prolonged CPR. This was attributed to circulatory mishap and unsuccessful CPR. However, in retrospect it was probably a result of increased PEEP and intrathoracic pressure. There is no possibility of validating this notion without a contemporary arterial blood gas analysis, and reports including cardiopulmonary monitoring as well as sonography during CPR are sparse.

We did not find any evidence indicating that the use of an automated CPR device is directly related to dynamic hyperinflation [19]. Assessment of out-in-the-field CPR by professional rescuers as well as animal and manikin CPR models have demonstrated that increased intrathoracic pressure most commonly results from excessive ventilation rates [20, 21]. In our case, ventilation frequency was predetermined by the ventilator at a rate of 12 breaths/minute for the most part of the prolonged CPR. However, in retrospect one may have to question whether the tidal volume of 10 ml/kilogram body weight – as supported by the current American Heart Association (AHA) and ERC guidelines – may have added to dynamic hyperinflation, especially since body weight may not be adequately assessed in the preclinical situation and during prolonged resuscitation.

Until more data are available intermittent disconnection of the respirator during prolonged CPR may be a reasonable strategy to avoid the possible fatal impact of increased intrathoracic pressure on cardiac preload and pulmonary perfusion.

## Conclusions

We agree that autoresuscitation events are probably under-reported [1, 13]. The under-reporting together with the low incidence of these events, hamper the elucidation of the pathophysiologic mechanisms involved. With this report and our critical review of the incident we hope to encourage other medical professionals to review and report their experiences.

Little is known about the hemodynamic impact of dynamic pulmonary hyperinflation during prolonged CPR. Dynamic hyperinflation of the lungs causes alveolar distention and collapse of small blood vessels, which results in increased dead space ventilation. The consequence is a drop in p_et_CO_2_, increased intrathoracic pressure, and obstruction of pulmonary blood flow precluding successful CPR. Continuous capnometry can easily be performed during CPR after endotracheal intubation. A drop in p_et_CO_2_ after prolonged CPR should always prompt reassessment of potential causes, such as: (i) failure to ventilate the lungs (for example displacement of the endotracheal tube), (ii) capnometer malfunction, and (iii) any other condition which occludes blood flow through the lungs. If massive hypovolemia and pulmonary artery embolism can be ruled out by emergent ultrasound, we suggest that disconnection of the ventilation may be a simple measure to resolve excessive PEEP and restore lung perfusion, which finally may allow for successful CPR.

## Abbreviations

AHA: American Heart Association
BUN: Blood urea nitrogen
CPPV: Continuous positive pressure ventilation
CPR: Cardiopulmonary resuscitation
CT: Computed tomography
ECG: Electrocardiogram
EMD: Electromechanical dissociation
ERC: European Resuscitation Council
FiO_2_: Fraction of inspired oxygen
IVC: Inferior vena cava
NSE: Neuron-specific enolase
PEEP: Positive end-expiratory pressure
p_et_CO_2_: End-tidal partial pressure of carbon dioxide
ROSC: Return of spontaneous circulation
VF: Ventricular fibrillation
